# Cross-Talk Between Pyroptosis and Ferroptosis Promotes Intestinal Inflammation and Barrier Failure During PEDV Infection

**DOI:** 10.3390/biom16050629

**Published:** 2026-04-23

**Authors:** Jie Peng, Wei-Gen Zhang, Hao Wang, Lin-Dong Qian, Ling-Bao Luo, Hong Gao, Xing-Neng Liu

**Affiliations:** 1 Department of Animal Husbandry and Veterinary Medicine, Yunnan Agricultural Vocational and Technical College, Kunming 650212, China; pengjie611@gmail.com (J.P.); ffe687159@gmail.com (L.-D.Q.); l13577842060@gmail.com (L.-B.L.); 2 College of Veterinary Medicine, Yunnan Agricultural University, Kunming 650201, China; weigeng1505@gmail.com; 3 College of Food Science and Technology, Yunnan Agricultural University, Kunming 650201, China; 2022110013@stu.ynau.edu.cn

**Keywords:** PEDV, pyroptosis, ferroptosis, intestine, multi-omics

## Abstract

Porcine epidemic diarrhea virus (PEDV) causes lethal enteritis in neonatal piglets, yet the mechanisms underlying rapid intestinal injury remain unclear. In particular, it is unknown whether different regulated cell death pathways act separately or cooperatively to worsen mucosal damage. To address this question, we performed multi-omics analyses of infected intestinal tissues and found concurrent activation of pyroptosis and ferroptosis during PEDV infection. PEDV infection activated the Caspa-se-1/GSDMD pathway in the duodenum and jejunum, as shown by generation of the Caspase-1 p20 fragment and cleavage of GSDMD into its active N-terminal form, indicating pyroptosis. At the same time, infected tissues displayed key features of ferroptosis, including weakened antioxidant defenses, increased lipid peroxidation, iron accumulation, lipid remodeling, and dysregulated ACSL4 and GPX4 expression. These two processes were closely linked and together contributed to tight junction disruption and barrier instability. Molecular docking further suggested that PEDV NSP1 and S proteins may interact with Caspase-1, providing a possible explanation for pyroptosis induction. Correlation analysis also showed strong associations between pyroptosis-related genes and ferroptosis-associated metabolites. Overall, our findings indicate that pyroptosis and ferroptosis cooperate to drive PEDV-induced intestinal inflammation and barrier damage, highlighting their joint inhibition as a potential strategy to reduce PEDV pathogenicity.

## 1. Introduction

Acute diarrhea caused by enteric viral infections remains a major threat to global livestock production and food security. The pathology, however, is not merely a consequence of viral replication, but rather reflects a system-level cascade-immune amplification of inflammation, epithelial barrier imbalance, and disrupted absorptive function-that culminates in diarrheal disease [1,2,3]. Porcine epidemic diarrhea virus (PEDV) is a highly pathogenic coronavirus that predominantly targets the enteric tract, exemplifying this threat [4] and causing explosive outbreaks of watery diarrhea and high mortality in neonatal piglets [5,6]. Its rapid evolution and capacity for immune evasion make control persistently challenging [7,8,9,10]. While villous epithelial injury in the small intestine is a pathological hallmark of PEDV infection [11,12], the precise molecular bridge linking initial viral insult to the ensuing catastrophic barrier failure remains poorly defined. In particular, whether distinct regulated cell death programs act independently or converge to dismantle the infected epithelium and propagate inflammation is a critical unresolved question.

Pyroptosis represents a form of lytic and inflammatory programmed cell death, which is triggered by the activation of inflammasome sensors and subsequently mediated by gasdermin family proteins. Upon activation, caspase-1 cleaves gasdermin D (GSDMD), thereby liberating its pore-forming N-terminal domain. This fragment then disrupts the integrity of the plasma membrane, facilitating the efflux of potent pro-inflammatory cytokines, including IL-1β and IL-18 [13,14]. Structural insights into the caspase-1–GSDMD interaction have illuminated the specificity of this cleavage event, providing a framework for understanding how pathogens might engage or evade this pathway [10,13,15]. In the context of PEDV, mitochondrial damage and the release of mitochondrial DNA have been observed in infected porcine intestinal epithelial cells, coinciding with NLRP3 inflammasome activation and IL-1β secretion [16]. Furthermore, PEDV is known to compromise mucosal integrity and downregulate tight-junction components [17]. Yet, whether these inflammasome signals progress beyond cytokine maturation to trigger the executioner phase of GSDMD-mediated pyroptosis in vivo, and whether this directly contributes to the profound barrier dysfunction seen in piglets, has remained unexplored.

The intestinal epithelium is also metabolically primed for another form of regulated cell death: ferroptosis. Its environment, rich in dietary iron and polyunsaturated fatty acids, creates a permissive setting for iron-catalyzed phospholipid peroxidation. This process is restrained by the system Xc^−^–GSH–GPX4 antioxidant axis and modulated by lipid-remodeling enzymes such as ACSL4; its hallmark features include the accumulation of lipid peroxides and distinct mitochondrial morphological changes [18,19,20]. Viral infections can subvert host metabolism to trigger ferroptosis, thereby shaping both inflammatory outcomes and viral fitness [21,22]. Furthermore, in vitro investigations into PEDV infection have identified reactive oxygen species (ROS) as key contributors to oxidative damage and as inducers of multiple distinct cell death pathways [23]. However, evidence demonstrating that ferroptosis is a causal driver of lipid peroxidation and barrier destabilization during enteric viral disease in vivo has been lacking. Critically, whether ferroptosis operates independently or in concert with the canonical caspase-1–GSDMD pyroptosis axis to amplify intestinal inflammation remains an open and important question.

Here we address this gap by investigating PEDV pathogenesis in a disease-relevant piglet model. Using an integrated multi-omics approach, we reveal that PEDV simultaneously activates both Caspase-1–GSDMD-mediated pyroptosis and a distinct ferroptosis program in the infected intestine. We uncover a cooperative interaction between these two death pathways that amplifies inflammation and drives tight-junction disruption, ultimately leading to barrier failure. Our findings establish a new paradigm of ‘pathogen cooperativity’ between pyroptosis and ferroptosis in the gut and identify their joint inhibition as a potential therapeutic strategy to preserve epithelial integrity and reduce PEDV pathogenicity.

## 2. Materials and Methods

### 2.1. Materials

The primary antibodies utilized in the present investigation were sourced from Proteintech (Wuhan, China), including Caspase-1 antibody (#22915-1-AP), GSDMD antibody (#20770-1-AP), JAM-1 antibody (#84720-2-RR), Occludin antibody (#27260-1-AP), ACSL4 antibody (#22401-1-AP), SLC7A11 antibody (#26864-1-AP), GPX4 antibody (#67763-1-Ig), NRF2 antibody (#16396-1-AP), FSP1 antibody (#20886-1-AP), and β-actin antibody (#66009-1-Ig). RT-qPCR reagents were purchased from YEASEN (Shanghai, China), including the MolPure^®^ Magnetic Tissue Total RNA Kit (#18605ES20) for RNA extraction, RT-gDNA Digestion SuperMix (#11142ES10) for reverse transcription, and qPCR SYBR Master Mix (#11184ES03) for amplification.

### 2.2. Animal Sample Collection

Intestinal samples were collected from 7- to 10-day-old piglets at a commercial farm in Yunnan, China, that exhibited clinical signs consistent with porcine epidemic diarrhea virus (PEDV) infection, including watery diarrhea, vomiting, and dehydration. PEDV infection was confirmed by RT-qPCR detection of the PEDV N gene in rectal swabs (Ct < 25). Only piglets that tested negative for transmissible gastroenteritis virus (TGEV), porcine deltacoronavirus (PDCoV), and porcine rotavirus (PoRV) by multiplex RT-PCR were included. Three age-matched healthy control piglets were selected from a farm with no history of PEDV infection or recorded outbreaks of enteric disease. Rectal swabs from control piglets tested negative for PEDV, TGEV, PDCoV, and PoRV by RT-qPCR, and these animals showed no clinical signs. Piglets in both groups were euthanized under deep anesthesia induced by intramuscular administration of Zoletil (50 mg/kg), followed by intravenous injection of potassium chloride (1–2 mg/kg). Intestinal tissues, including the duodenum, jejunum, and ileum, were collected immediately after euthanasia and flushed with ice-cold PBS to remove luminal contents. The samples were then snap-frozen in liquid nitrogen and stored at −80 °C for 3 to 6 months.

### 2.3. RNA-Seq Analysis of PEDV-Infected Intestinal Samples

The duodenum samples from the piglets were sent to Aptbiotech Co., Ltd (Shanghai, China). for RNA-seq analysis. RNA-seqing was conducted on the Illumina NovaSeq 6000 platform (Illumina, Inc., San Diego, CA, USA). Gene expression quantification was conducted using featureCounts (2.0.3), and differential expression analysis was performed with DESeq2. The selection criteria for differentially expressed genes (DEGs) were |log2FC| ≥ 1.3 and a corrected *p* value (P_adj) < 0.05. Kyoto Encyclopedia of Genes and Genomes (KEGG) pathway enrichment and Gene Ontology (GO) functional annotation were performed for the identified DEGs.

### 2.4. RT-qPCR Quantification of mRNA Expression

mRNA levels of *NLRP3*, *caspase-1*, *GSDMD*, and *IL-1β*, which are key markers of inflammasome-mediated pyroptosis, were quantified in intestinal tissues by RT-qPCR as previously described [24]. TNF-α was also measured as a representative proinflammatory cytokine to assess the broader intestinal inflammatory response. *GAPDH* was used as the internal reference gene. The mRNA expression levels were calculated using the online tool “qPCR Data Analysis” (https://hiplot.com.cn/shiny2/QPCR_Analysis/QPCR_Analysis/, 7 November 2025, Shanghai, China). Primer sequences are provided in Supplementary Table S1.

### 2.5. Quantification of Protein Expression

Proteins were extracted from duodenal and jejunal tissues stored at −80 °C. Briefly, 80 mg of tissue was homogenized for 3 min in RIPA lysis buffer (#P0013B, Beyotime, Shanghai, China) supplemented with protease inhibitor cocktail (#04693159001, Roche, Basel, Switzerland) using a tissue homogenizer. The homogenates were then incubated on ice for 30 min, with vortexing every 10 min, and centrifuged at 12,000× *g* for 15 min at 4 °C. The supernatants were collected, and protein concentrations were determined using a BCA protein assay kit (#23225, Thermo, Waltham, MA, USA). Protein expression levels were analyzed by Western blotting after SDS-PAGE and electrophoretic transfer to membranes, as previously described [25]. The membranes were incubated with the corresponding primary antibodies against caspase-1, GSDMD, occludin, JAM-1, ACSL4, SLC7A11, GPX4, Nrf2, β-actin, and FSP1.

### 2.6. Molecular Docking

The three-dimensional structures of Caspase-1 and PEDV-related proteins were obtained from the Protein Data Bank (RCSB PDB, https://www.rcsb.org/, 11 November 2025). The corresponding PDB IDs are: Caspase-1 (1BMQ), PEDV-N (8WQK), PEDV-S (7Y6U), PEDV-nsp1 (5XBC), PEDV-nsp4 (8XPI), and PEDV-nsp9 (5HIZ). After obtaining these protein structures, molecular docking was subsequently carried out using the HDOCK server to investigate potential interactions (http://hdock.phys.hust.edu.cn/, 13 November 2025, Wuhan, China). A docking score lower than −5 (with scores of −200 or greater) was considered indicative of a potential binding interaction. A confidence score above 0.7 was used as the threshold to suggest a high probability of interaction between the molecules [26,27].

### 2.7. Metabolomic Analysis of PEDV-Infected Intestinal Samples

Metabolites in intestinal tissues were analyzed using untargeted metabolomics techniques. Mass spectrometry analysis was conducted by Biomarker Technologies (Beijing, China) utilizing a Q-Exactive HF-X high-resolution mass spectrometer, operating under both positive and negative ion modes. The annotation of metabolites was performed based on the HMDB database. Differential metabolites (DMs) were identified by Student’s *t* test with a threshold of VIP > 1 and *p* < 0.05 and FDR correction.

### 2.8. Lipid Peroxidation and Iron Assays

Commercial kits were used to assess oxidative stress markers, including GSH-Px (#A005-1-2, Nanjing Jiancheng, Nanjing, China) and MDA (#AKFA013M, Boxbio, Beijing, China). All procedures followed the manufacturer’s instructions.

Ferrous iron (Fe^2+^) concentration in intestinal tissues was measured using an Fe^2+^ detection kit (#E-BC-K773-M, Elabscience, Wuhan, China), according to the manufacturer’s instructions. Briefly, intestinal tissues were homogenized in assay buffer, followed by centrifugation and collection of the supernatant. The supernatant was mixed with the working solution, incubated at 37 °C in the dark for the prescribed duration, and absorbance was measured at the designated wavelength using a microplate reader.

### 2.9. Data Statistical Analysis

Statistical analyses were performed using GraphPad Prism 10.0. Data normality was assessed with the Shapiro–Wilk test, and homogeneity of variance was evaluated using Levene’s test. Differences between groups were analyzed using Student’s *t*-test.

### 2.10. AI Clarification

During the preparation of this manuscript, ChatGPT 5.4 was utilized for language translation and text polishing. Following the use of this tool, the authors conducted a thorough review and made necessary revisions to the content, and assume full responsibility for the final version of the published article.

## 3. Results

### 3.1. The Pyroptosis Pathway Regulated by Caspase-1/GSDMD Is Involved in PEDV Infection

**Figure 1:**
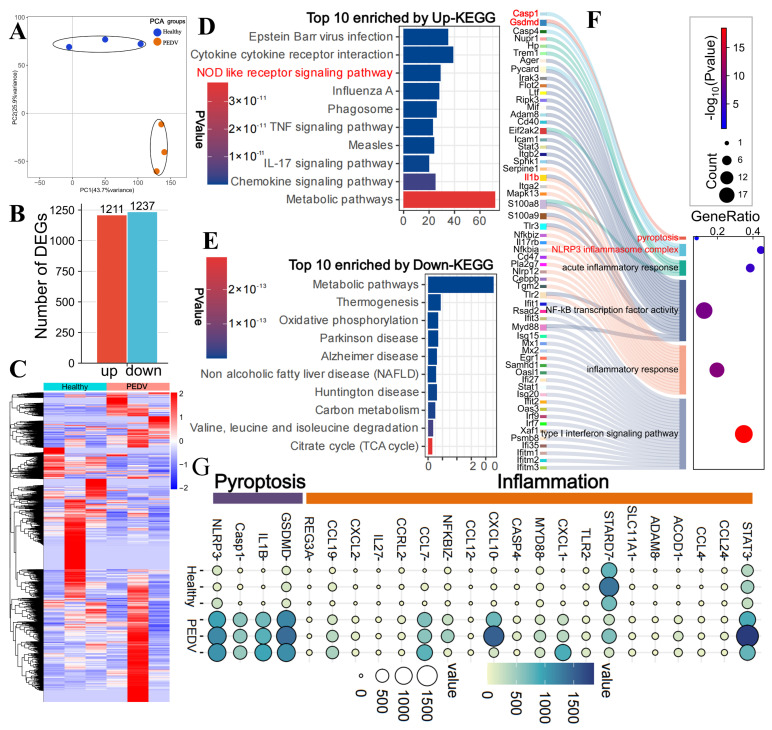
Caspase-1/GSDMD Pyroptosis Pathway in PEDV Infection. (**A**) Principal component analysis (PCA) of transcriptomic data showing clear separation between groups. (**B**) Statistical analysis of DEGs between groups. (**C**) Heatmap illustrating the expression patterns of DEGs (red—upregulation, blue—downregulation). (**D**) KEGG pathway enrichment analysis of upregulated DEGs (Top 10). (**E**) KEGG enrichment analysis of downregulated DEGs (Top 10). (**F**) GO enrichment analysis of upregulated DEGs. (**G**) Heatmap analysis of pyroptosis and inflammation associated DEGs.

To investigate the molecular mechanisms underlying intestinal damage during PEDV infection, RNA-seq was performed on intestinal tissues from infected pigs. As shown in Figure 1A, principal component analysis (PCA) revealed a clear separation between the infected and control groups, indicating significant alterations in the transcriptional profile of intestinal tissues following PEDV infection. Differential expression analysis identified 2448 DEGs, with 1211 genes upregulated and 1237 downregulated (Figure 1B). The heatmap of DEGs further confirmed that PEDV infection notably reshaped the gene expression profile of the jejunum (Figure 1C).

Subsequent KEGG enrichment analysis of the upregulated and downregulated genes revealed a significant enrichment of the NOD-like receptor signaling pathway in the upregulated gene set [28], suggesting that PEDV infection may activate inflammasome-related pathways (Figure 1D,E). Additionally, multiple classical inflammatory pathways, including the TNF and IL-17 pathways, were significantly enriched in the upregulated genes, indicating widespread inflammation induced by PEDV infection (Figure 1D,E). GO analysis of the upregulated genes in the NOD-like receptor signaling and related inflammation pathways further identified the activation of the Caspase-1/GSDMD pathway, as well as enrichment of the NF-κB signaling pathway (Figure 1F). Consistent with these findings, differential expression analysis revealed significant upregulation of key pyroptotic molecules, including NLRP3, Caspase-1, GSDMD, and IL-1β, alongside increased expression of multiple inflammatory genes (Figure 1G). Taken together, transcriptomic and gene expression analyses strongly support the activation of the Caspase-1/GSDMD pathway during PEDV infection, which may contribute to intestinal damage.

### 3.2. Activation of Caspase-1/GSDMD Pathway in PEDV Infection

**Figure 2:**
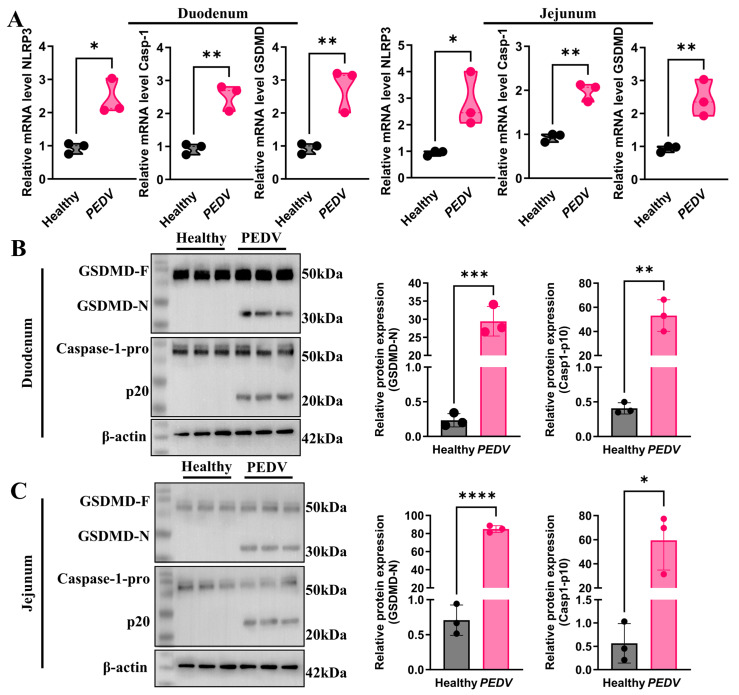
Activation of Caspase-1/GSDMD Pathway in PEDV Infection. (**A**) qRT-PCR analysis of the mRNA levels of *NLRP3*, *Caspase-1*, and *GSDMD* in the jejunum and ileum of PEDV-infected and healthy control piglets (*n* = 3); (**B**) Western blot analysis of Caspase-1 and GSDMD protein expression and activation in the jejunum, accompanied by quantification of their cleaved (activated) fragments (*n* = 3, Original Western blot images can be found in Figure S1A); (**C**) Western blot analysis of Caspase-1 and GSDMD protein levels and activation in the ileum, with quantification of cleaved (activated) fragments (*n* = 3, Original Western blot images can be found in Figure S1B). Data are presented as mean ± SEM (Student’s *t*-test). * *p* < 0.05, ** *p* < 0.01, *** *p* < 0.001, **** *p* < 0.0001.

To further investigate the role of the Caspase-1/GSDMD pathway in PEDV infection, qRT-PCR was performed to assess the mRNA expression levels of *NLRP3*, *Caspase-1*, and *GSDMD* in the jejunum tissues of healthy controls and PEDV-infected pigs, with GAPDH used as an internal control. As shown in Figure 2A, PEDV-infected pigs exhibited significantly elevated mRNA expression levels of *NLRP3*, *Caspase-1*, and *GSDMD* in the jejunum compared with the control group. A similar trend was observed in the ileum tissues of infected pigs.

To evaluate activation of the Caspase-1/GSDMD pathway, Western blot analysis was conducted on jejunal tissue samples. The results demonstrated that PEDV infection markedly enhanced Caspase-1 activation and increased the generation of the cleaved Caspase-1-p20 fragment. Moreover, activated Caspase-1 cleaved GSDMD to generate its N-terminal fragment (GSDMD-N). (Figure 2B). A similar cleavage pattern and pathway activation were observed in the ileum tissues (Figure 2C). These results suggest that PEDV infection activates the Caspase-1/GSDMD signaling axis in intestinal tissues, leading to pyroptosis in intestinal epithelial cells.

### 3.3. Molecular Docking Prediction of Caspase-1 Binding with PEDV-Related Proteins

**Figure 3:**
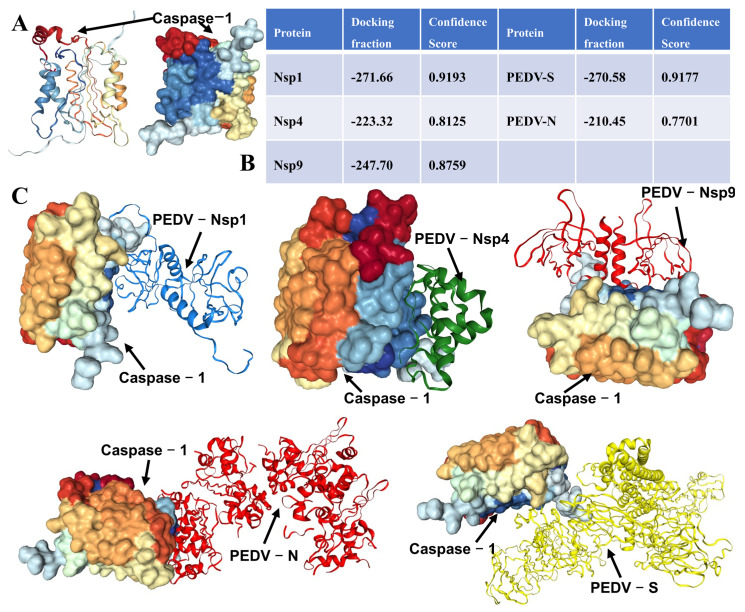
Molecular Docking Prediction of Caspase-1 Binding with PEDV-Related Proteins. (**A**) Three-dimensional structure of Caspase-1. (**B**) Docking scores and confidence for Caspase-1 binding with PEDV-related proteins. (**C**) Predicted docking interactions between Caspase-1 and PEDV-related proteins.

To elucidate the potential mechanism by which PEDV activates the Caspase-1/GSDMD pathway, molecular docking was performed to assess the interactions between Caspase-1 and key viral proteins, including PEDV-Nsp1, PEDV-Nsp4, PEDV-Nsp9, PEDV-S, and PEDV-N. Results from HDOCK analysis indicated that Caspase-1 binds with all these PEDV proteins (Figure 3A,B). Among them, Caspase-1 exhibited the strongest binding affinities with PEDV-Nsp1 and PEDV-S, with docking scores of −271.66 and −270.58, respectively (Figure 3B). The optimal docking conformations of Caspase-1 with each PEDV-related protein are depicted in Figure 3C. These findings suggest that PEDV may interact with Caspase-1 through PEDV-Nsp1 and PEDV-S, thereby promoting the activation of the Caspase-1/GSDMD-mediated pyroptosis pathway.

### 3.4. PEDV Infection Exacerbates Intestinal Oxidative Stress and Lipid Peroxidation

**Figure 4:**
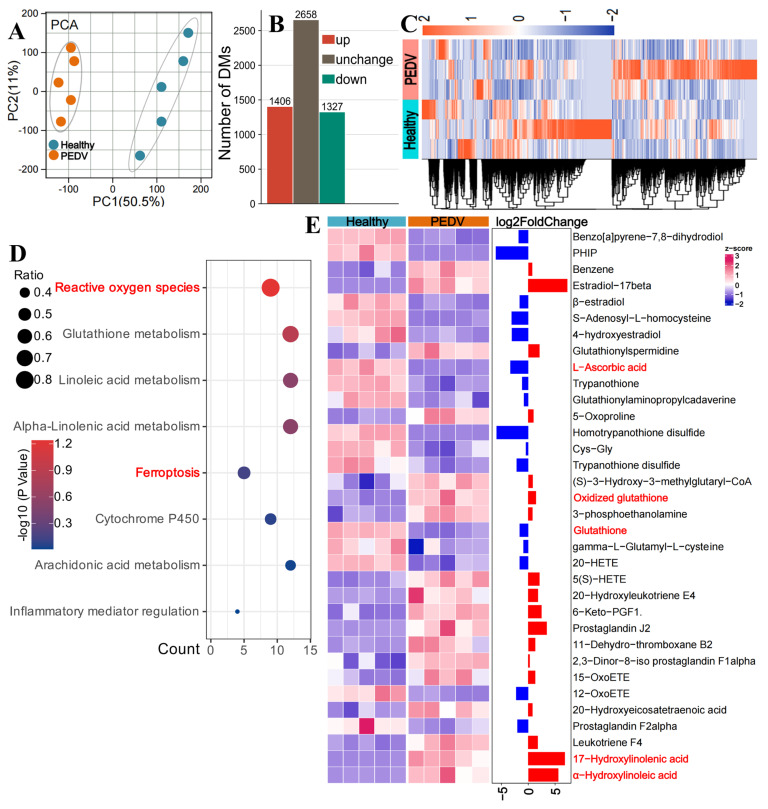
PEDV Infection Exacerbates Intestinal Oxidative Stress and Lipid Peroxidation. (**A**) PCA showing significant differences in metabolite profiles between groups. (**B**) Statistical analysis of differential metabolites between PEDV-infected and healthy control groups. (**C**) Heatmap of metabolite profiles in the intestines of healthy and PEDV-infected piglets. (**D**) KEGG pathway enrichment of DMs, highlighting pathways related to oxidative stress, lipid peroxidation, and ferroptosis, including lipid metabolism, ROS metabolism, and arachidonic acid metabolism. (**E**) Clustering analysis of metabolites involved in oxidative stress, lipid peroxidation, and ferroptosis.

To investigate alterations in oxidative stress and lipid peroxidation during PEDV infection, metabolomic profiling was performed on intestinal samples collected from healthy and PEDV-infected piglets. PCA revealed a clear separation in metabolite composition between the two groups (Figure 4A). Differential metabolite analysis identified 1406 upregulated metabolites and 1327 downregulated metabolites in the PEDV-infected group compared to controls (Figure 4B). Cluster analysis further demonstrated that PEDV infection significantly altered the overall metabolic profile of the intestinal tissues (Figure 4C).

KEGG enrichment analysis of the differential metabolites (DMs) (Figure 4D) demonstrated significant enrichment in pathways related to reactive oxygen species, glutathione metabolism, linoleic acid metabolism, α-linolenic acid metabolism, and arachidonic acid metabolism, all of which are closely linked to oxidative stress and lipid peroxidation processes [29]. Additionally, PEDV infection significantly enriched metabolites in the ferroptosis pathway. Further clustering analysis of metabolites associated with oxidative stress and lipid peroxidation regulation in ferroptosis (Figure 4E, Supplementary Table S2) indicated that PEDV infection reduced the levels of antioxidants and lipid peroxidation inhibitors, such as glutathione and L-ascorbic acid, while increasing metabolites that promote oxidative stress and lipid peroxidation, including oxidized glutathione, 17-hydroxylinolenic acid, and α-hydroxylinoleic acid [30]. These results suggest that ferroptosis may play a role in the pathogenesis of PEDV infection.

### 3.5. PEDV Infection Activates Intestinal Cell Ferroptosis

**Figure 5:**
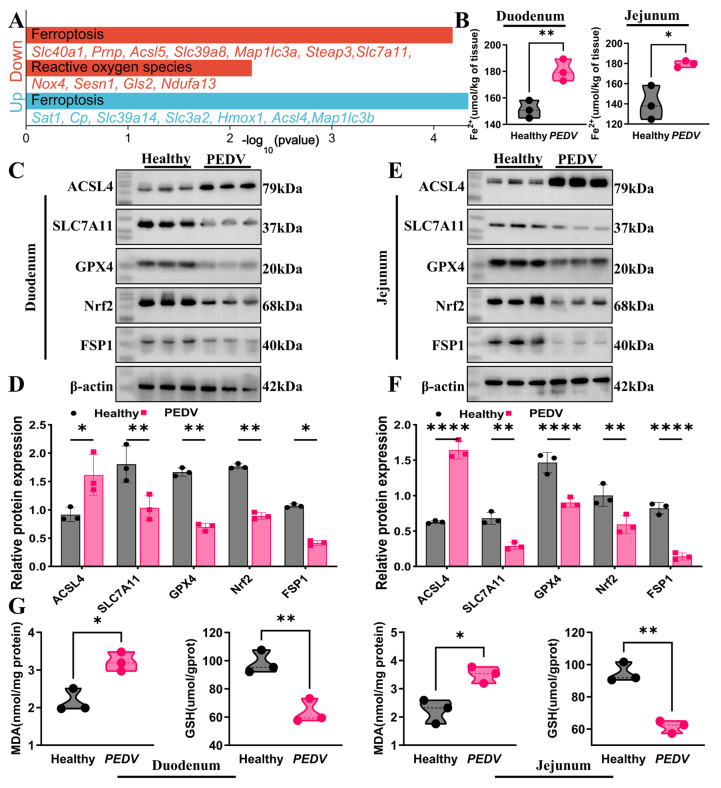
PEDV Infection Induces Ferroptosis in Intestinal Cells. (**A**) KEGG enrichment analysis of ferroptosis- and reactive oxygen species-related genes was visualized based on RNA-sequencing data. Compared with the healthy control group, these genes were significantly differentially expressed in PEDV-infected tissues. (**B**) Measurement of ferrous iron (Fe^2+^) levels in the jejunum and ileum of PEDV-infected and control piglets (*n* = 3). (**C**,**D**) Western blot analysis was performed to determine the expression of ferroptosis-associated proteins (ACSL4, SLC7A11, GPX4, Nrf2, and FSP1) in the jejunum, and protein levels were quantitatively analyzed (*n* = 3, Original Western blot images can be found in Figure S2A). (**E**,**F**) Western blot analysis was conducted to assess the expression of ferroptosis-related proteins in the ileum, followed by quantitative evaluation of protein levels (*n* = 3, Original Western blot images can be found in Figure S2B). (**G**) Analysis of lipid peroxidation markers (GSH-Px and MDA) in intestinal tissues (*n* = 3). Data are presented as mean ± SEM (Student’s *t*-test). * *p* < 0.05, ** *p* < 0.01, **** *p* < 0.0001.

To clarify the molecular basis by which PEDV infection triggers ferroptosis, we visualized the genes enriched in the Ferroptosis pathway from RNA-seq analysis. The results indicated significant enrichment of typical regulatory genes associated with Ferroptosis. Notably, PEDV infection increased ACSL4 expression while reducing the expression of SLC7A11 (Figure 5A). The accumulation of iron ions, particularly Fe^2+^, is a key driver of Ferroptosis [31,32]. Compared with healthy controls, PEDV infection markedly elevated Fe^2+^ levels in both the jejunal and ileal tissues (Figure 5B).

Further examination of Ferroptosis markers revealed that PEDV infection notably altered the expression profiles of these markers. In the jejunum, ACSL4 expression was upregulated, while the expressions of SLC7A11, GPX4, Nrf2, and FSP1 were significantly downregulated (Figure 5C,D). These changes were similarly observed in the ileum tissues of PEDV-infected piglets (Figure 5E,F). Additionally, oxidative stress and lipid peroxidation are core biochemical processes that characterize Ferroptosis. Consequently, we assessed the levels of oxidative stress and lipid peroxidation indicators during PEDV infection. As shown in Figure 5G, PEDV infection significantly increased MDA levels and GSH-Px activity in the jejunum and ileum, exacerbating oxidative stress and lipid peroxidation. These findings indicate that PEDV infection markedly promotes Ferroptosis in intestinal tissues.

**Figure 6:**
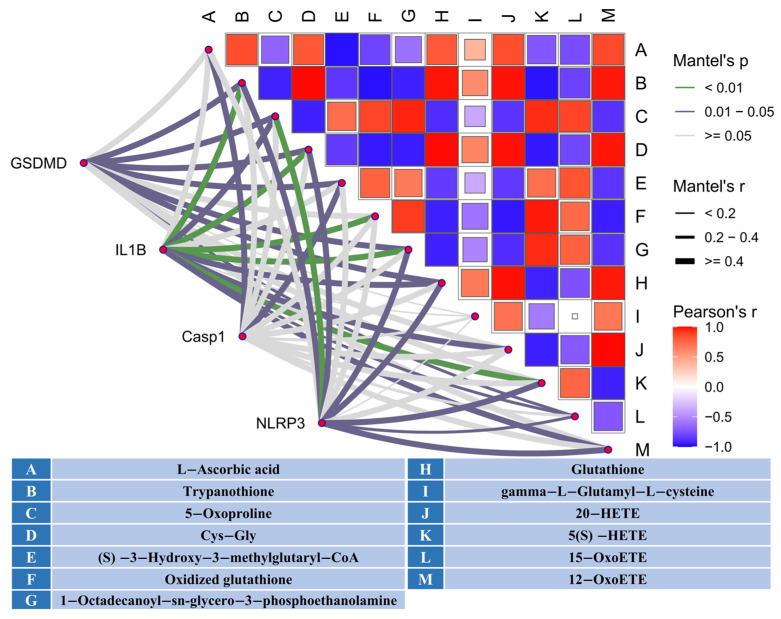
Correlation Between Caspase-1/GSDMD-Mediated Pyroptosis and Ferroptosis-Related Metabolites (Mantel Test). Matrix on the right shows Pearson correlation (represented by color intensity) between ferroptosis-related metabolites. Mantel’s test was employed to assess the correlation between pyroptosis-related gene expression and metabolites.

To further explore the potential connection between pyroptosis and Ferroptosis during PEDV infection, we conducted a Mantel test to correlate genes involved in pyroptosis with metabolites associated with Ferroptosis. The analysis revealed a significant correlation between genes mediating pyroptosis and metabolites regulating Ferroptosis (Mantel’s *p* ≤ 0.05 and Mantel’s r ≥ 0.4) (Figure 6), suggesting a potential synergistic regulation between these two forms of cell death during PEDV infection.

### 3.6. PEDV Infection Exacerbates Intestinal Inflammation and Disrupts Tight Junctions

**Figure 7:**
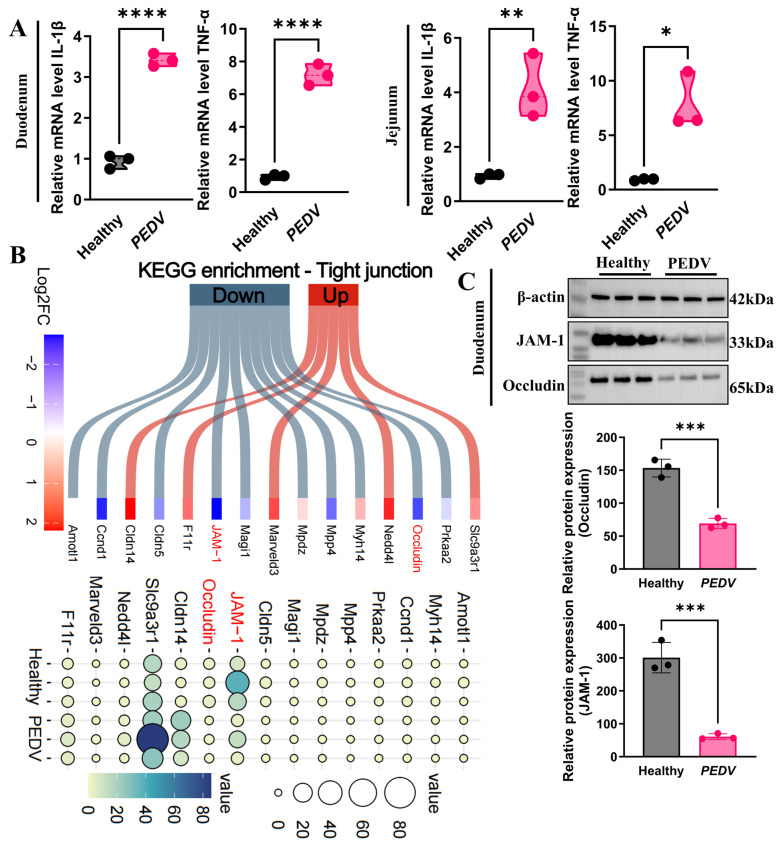
PEDV Infection Exacerbates Intestinal Inflammation and Disrupts Tight Junction Integrity. (**A**) qRT-PCR analysis of *IL-1β* and *TNF-α* mRNA levels in the jejunum and ileum of PEDV-infected and healthy control piglets (*n* = 3); (**B**) KEGG pathway enrichment analysis of tight junction-related genes following PEDV infection (Original Western blot images can be found in Figure S2C); (**C**) Western blot analysis of JAM-1 and Occludin protein levels in the jejunum, with quantification of protein expression (*n* = 3). Data are presented as mean ± SEM (Student’s *t*-test). * *p* < 0.05, ** *p* < 0.01, *** *p* < 0.001, **** *p* < 0.0001.

Pyroptosis is a pro-inflammatory mode of cell death that is generally associated with pronounced inflammatory responses [33,34]. To assess the intestinal inflammatory response after PEDV infection, we quantified the expression levels of pro-inflammatory cytokines. As shown in Figure 7A, the levels of *IL-1β* and *TNF-α* in both the jejunum and ileum of piglets infected with PEDV were markedly elevated relative to those observed in the control group. These results suggest that PEDV infection markedly promotes the production of inflammatory cytokines in the intestine, thereby aggravating intestinal inflammation.

During PEDV infection, the induced pyroptosis and ferroptosis pathways often lead to epithelial cell death, which subsequently disrupts the integrity of the intestinal epithelial tight junctions [35,36]. To investigate this further, we conducted KEGG pathway enrichment analysis of tight junction-related genes in the transcriptome data from the PEDV-infected group. The analysis revealed that PEDV infection significantly altered the expression of tight junction pathway-related genes, with both upregulated and downregulated genes involved. Specifically, the mRNA levels of key tight junction proteins, including *JAM-1* and *Occludin*, were significantly reduced following PEDV infection (Figure 7B). Protein expression analysis further confirmed that PEDV infection led to a significant decrease in the levels of JAM-1 and Occludin in jejunal tissue (Figure 7C). Collectively, these findings indicate that PEDV infection impairs tight junction integrity by suppressing the expression of critical tight junction components (JAM-1 and Occludin), thereby compromising intestinal barrier function.

## 4. Discussion

This study shows that PEDV infection in the intestines of piglets induces both Caspase-1/GSDMD-dependent pyroptosis and iron-dependent lipid peroxidation-driven ferroptosis, and suggests that these pathways cooperate to promote intestinal inflammation and barrier failure. RNA-seq revealed marked enrichment of the NOD-like receptor signaling pathway, accompanied by increased expression of NLRP3, CASP1, GSDMD, and IL-1β, supporting activation of the inflammasome pathway. Activation of caspase-1, as indicated by its cleavage to caspase-1 p20, subsequently promotes GSDMD processing and generation of the pyroptotic fragment GSDMD-N, a hallmark of pyroptosis [37,38]. Detection of cleaved caspase-1 p20 and GSDMD-N further indicates activation of the pore-forming execution program of pyroptosis. These findings are consistent with the established role of gasdermin family proteins in membrane permeabilization and inflammatory mediator release during infection-associated inflammation [7,31]. They also agree with structural evidence showing that GSDMD pores mediate the secretion of mature IL-1 family cytokines [7,31]. Previous in vitro work in PEDV models linked mitochondrial injury and mtDNA release to activation of the NLRP3–IL-1β axis [16]. Our in vivo data extend this framework by connecting Caspase-1 activation and GSDMD cleavage to intestinal inflammation and barrier dysfunction, thereby linking molecular execution events to pathological outcome. However, because the present analyses largely reflect whole-tissue responses, they do not resolve the relative contributions of epithelial cells and innate immune populations. Future studies integrating spatial transcriptomics and single-cell RNA sequencing should define the cellular sources and context of GSDMD-N expression with greater precision.

Evidence for ferroptosis was similarly compelling. Upregulation of ACSL4, together with downregulation of SLC7A11, GPX4, Nrf2 and FSP1, accompanied by Fe^2+^ accumulation, increased MDA and depleted GSH, indicates collapse of the system Xc^−^–GSH–GPX4 antioxidant axis and activation of a canonical ferroptotic program characterized by enhanced PUFA-phospholipid vulnerability, lipid peroxide accumulation and membrane damage [18,39]. This mechanism is particularly relevant to the intestinal epithelium, which is continuously exposed to oxidative stressors and dietary iron and is therefore highly dependent on redox buffering and lipid homeostasis. Consistent with this interpretation, ferroptosis has been implicated in epithelial dysfunction across multiple intestinal disorders, and tight junctions are especially sensitive to oxidative and inflammatory injury [40,41]. Notably, the metabolomic profile revealed increased antioxidant metabolites alongside enhanced oxidative damage, a pattern more consistent with incomplete compensatory mobilization than with effective suppression of ferroptotic stress.

A central implication of these findings is that pyroptosis and ferroptosis may not represent independent responses to PEDV infection, but rather interconnected components of a broader pathogenic program. Integration of transcriptomic, metabolomic and histopathological data supports coordinated activation of these pathways. One plausible point of convergence is the mitochondrial ROS–DAMP axis, which can potentiate inflammasome activation [42]. At the same time, lipid peroxidation and disrupted iron homeostasis may increase membrane fragility, thereby lowering the threshold for GSDMD-mediated membrane damage [14]. In this context, NLRP3 may serve as a signaling hub linking distinct inflammatory death programs [43]. Studies in coronavirus models have shown that suppression of inflammasome/pyroptotic signaling can be offset by alternative inflammatory death pathways, including Caspase-8- or RIPK3-dependent necroptosis, highlighting the plasticity of infection-triggered death networks [43]. On the basis of the present data, we propose a pyroptosis–ferroptosis synergy model in which inflammatory lysis and lipid peroxidation act together to drive epithelial barrier collapse and diarrheal disease. This framework also provides a rationale for combinatorial intervention: suppressing Caspase-1/GSDMD activity to limit IL-1β-associated barrier leakage, while simultaneously restraining iron-dependent lipid peroxidation to preserve epithelial resilience.

The close alignment between Caspase-1/GSDMD activation and the intestinal injury phenotype further supports this model. Caspase-1 activation facilitates the maturation of IL-1β, while PEDV infection upregulates additional inflammatory mediators, including TNF-α. These combined processes synergistically disrupt epithelial homeostasis within the local tissue microenvironment [44,45]. In parallel, both transcript and protein analyses showed reduced expression of the tight-junction components occludin and JAM-1 [44,45]. A parsimonious interpretation is that GSDMD-N pore formation not only drives cell swelling and lysis but also contributes, directly or indirectly, to junctional destabilization. In addition, IL-1β and TNF-α may activate downstream pathways such as myosin light chain kinase, thereby promoting internalization and degradation of tight-junction proteins, as described in models of intestinal inflammation [46]. Although the present molecular docking analysis suggests possible interactions between PEDV Nsp1 or S proteins and Caspase-1, these data are insufficient to support a conclusion that the virus directly hijacks this target. Such a model will require orthogonal validation, including co-immunoprecipitation, in situ proximity ligation assays, interactome proteomics and interface-disrupting mutagenesis. Likewise, definitive assessment of causality in vivo will depend on perturbation studies. Combining Caspase-1 inhibition, for example with VX-765, or GSDMD-targeting approaches with ferroptosis blockade will be necessary to determine whether dual-pathway targeting confers synergistic protection against diarrhea, inflammatory output and tight-junction loss.

We acknowledge several limitations of this study. First, the sample size was relatively small, reflecting adherence to the 3Rs principle in a large-animal experimental setting. Although preliminary power analysis indicated sufficient power to detect large effect sizes and the observed significant differences were robust, larger cohort studies are needed to confirm these findings and assess inter-individual variability. Second, although our multi-omics data provide strong correlative evidence for crosstalk between pyroptosis and ferroptosis, direct cellular evidence and functional validation using specific pathway inhibitors are required to establish causality. Future studies should employ transmission electron microscopy to identify the characteristic ultrastructural features of pyroptosis, including GSDMD pore formation and cellular swelling, and ferroptosis, including shrunken mitochondria with increased membrane density and loss of cristae, in PEDV-infected enterocytes. Furthermore, selective small-molecule inhibitors targeting caspase-1 or ferroptosis, applied either in vivo or in PEDV-infected porcine intestinal epithelial cells (IPEC-J2), will help determine whether these pathways directly drive the observed cell death and barrier dysfunction. Such experiments will also help evaluate the therapeutic potential of dual-pathway inhibition in reducing PEDV pathogenicity.

In summary, these findings move the pathogenic model of PEDV beyond a simple replication–inflammation framework towards one in which inflammatory cell lysis and lipid peroxidation cooperate to drive intestinal barrier failure. Future work should resolve the temporal order and cellular interdependence of pyroptosis and ferroptosis and use causal loss-of-function strategies to define their relative contributions to diarrheal pathogenesis. Such studies may lay the groundwork for mechanism-based anti-diarrheal interventions that target both pathways simultaneously.

## 5. Conclusions

This study demonstrates that PEDV infection induces intestinal injury in piglets through a dual mechanism involving pyroptosis and ferroptosis. PEDV activates the Caspase-1/GSDMD-mediated pyroptosis pathway while simultaneously promoting ferroptosis, thereby exacerbating oxidative stress and lipid peroxidation. The close association between pyroptosis-related genes and ferroptosis-associated metabolites suggests that these two cell death pathways may act synergistically to drive intestinal damage. Collectively, these findings offer new insights into PEDV pathogenesis and establish a theoretical basis for the development of combinatorial therapeutic strategies.

## Data Availability

The original contributions presented in this study are included in the article/Supplementary Material. Further inquiries can be directed to the corresponding author.

## Supplementary Materials

The following supporting information can be downloaded at https://www.mdpi.com/article/10.3390/biom16050629/s1, Table S1: Primers used for qPCR analysis, Table S2: Differential metabolic values and fold change, Figure S1: Original Western blot images for Figure 2B,C, Figure S2: Original Western blot images for Figure 5C,E and Figure 7C.

## Abbreviations

ACSL4	Acyl-CoA synthetase long chain family member 4
Caspase-1	Cysteine-aspartic acid protease 1
DEGs	Differentially expressed genes
DMs	Differential metabolites
FSP1	Ferroptosis-suppressor-protein 1
GSDMD	Gasdermin D
GO	Gene Ontology
GSH-Px	Glutathione Peroxidase
GPX4	Glutathione Peroxidase 4
JAM-1	Junctional adhesion molecule 1
KEGG	Kyoto Encyclopedia of Genes and Genomes
IL-1β	Interleukin-1 beta
IL-18	Interleukin 18
MDA	Malondialdehyde
Nrf2	Nuclear factor erythroid 2-related factor 2
NLRP3	NOD-like receptor thermal protein domain associated protein 3
PEDV	Porcine epidemic diarrhea virus
PCA	Principal component analysis
ROS	Reactive oxygen species
SLC7A11	Solute carrier family 7 member 11
TNF-α	Tumor necrosis factor-alpha
