# Supplementary material for: Cross-Talk Between Pyroptosis and Ferroptosis Promotes Intestinal Inflammation and Barrier Failure During PEDV Infection
Source: Biomolecules. 2026 Apr 23;16(5):629. doi: 10.3390/biom16050629 (PMC13204265; doi:10.3390/biom16050629)
Supplement: Supplementary file 1 [file biomolecules-16-00629-s001.zip › biomolecules-4240601-supplementary.pdf]

Supplementary Material

Cross-Talk Between Pyroptosis and Ferroptosis Promotes Intestinal Inflammation and Barrier Failure During PEDV Infection

Jie Peng [1,†], Wei-Gen Zhang [2,†], Hao Wang [3], Lin-Dong Qian [1], Ling-Bao Luo [1], Hong Gao [2,*] and Xing-Neng Liu [1,*]

Table S1 Primers used for qPCR analysis.

Genes	Primer sequences	Genes	Primer sequences
NLRP3	GATTATGGTTGGGACAGTGT	*IL-1β*	CAGTCTATGGAGTGCAAACT
NM_001256770.2	GACCAACCAGAGCTTCTTTA	NM_214055.1	GGTCACAGGTATCTTGTTGT
Casp-1	GCTATAGAGCTGAAGGCATT	*TNF-α*	GATCATCGTCTCAAACCTCA
NM_214162.1	AAACTTGGACAGTTCAAGGT	JF831365.1	TGGGAGTAGATGAGGTACAG
GSDMD	CTTTGAGAGGGTGGTCAAG	*GAPDH*	GATGACATCAAGAAGGTGGT
XM_021090506.1	ACAGGTCAACACATGTGTAG	NM_001206359.1	AAAGTGGAAGAGTGAGTGTC

Table S2 Differential metabolic values and fold change.

group	healthy	healthy	healthy	healthy	healthy	PEDV	PEDV	PEDV	PEDV	PEDV	log2FoldChange	
**Metabolite**	**healthy-1**	**healthy-2**	**healthy-3**	**healthy-4**	**healthy-5**	**PEDV-1**	**PEDV-2**	**PEDV-3**	**PEDV-4**	**PEDV-5**	**log2FoldChange**	
Benzo[a]pyrene-7,8-dihydrodiol	302.122225	305.64939	300.5361326	341.614038	352.654301	90.775499	108.904684	117.6384194	66.24227633	73.204588	-1.810867373	down
PHIP	192.830913	170.71155	284.8106901	173.571924	206.327341	1.0077556	0.085511	3.838995833	6.552053156	3.859501945	-6.066393184	down
Benzene	205.630456	200.45666	176.9821829	263.173658	203.045075	357.10349	332.042653	410.145469	331.5489415	328.4216521	0.745559764	up
Estradiol-17beta	8.20586532	10.843489	25.60600639	7.75083899	22.309957	3178.1302	2219.87884	3039.212717	1396.509498	1968.602778	7.303436225	up
β-estradiol	9063.20279	14549.325	11088.6967	13166.0595	11278.9481	3229.9626	4687.22974	4737.920704	3571.388474	3190.053277	-1.606999031	down
S-Adenosyl-L-homocysteine	7002.9995	7218.6204	8354.200052	8987.92921	9059.94806	756.94752	614.161167	776.9655217	898.9186611	1618.957755	-3.122078601	down
4-hydroxyestradiol	208.944438	494.28152	406.838552	770.927217	921.671385	60.321225	71.9406638	154.8129622	44.87947482	1.36E-09	-3.07774168	down
Glutathionylspermidine	54.0987223	89.570725	257.9397185	87.6606436	209.798405	615.77417	833.970258	520.7740291	473.7372535	534.7598457	2.091330512	up
L-Ascorbic acid	4786.03909	4033.9923	5629.783125	3786.36624	3921.93707	183.90107	351.679683	513.9891512	279.2939689	548.1297138	-3.351460204	down
Trypanothione	3077.2163	3129.529	3284.320687	3409.85809	2814.5489	1644.4418	1297.82878	1005.921027	1625.126584	1583.789353	-1.134737117	down
Glutathionylaminopropylcadaverine	699.023068	1055.5624	977.0743753	1001.91483	986.160718	532.32632	500.534251	557.3816298	674.8310686	400.4317355	-0.824297084	down
5-Oxoproline	3854.03109	3763.9427	4665.598096	4488.72012	4233.42372	6284.719	10198.1739	10016.10818	7430.832844	7550.023055	0.981628973	up
Homotrypanothione disulfide	4584.22717	4170.6025	6158.741608	6219.12207	5895.7829	44.910542	99.4837552	21.47453076	171.6609891	113.4008803	-5.905430644	down
Cys-Gly	854.22616	845.5199	845.4228735	741.490364	854.551234	608.8605	554.727224	536.1532452	630.6038503	694.8034273	-0.453046707	down
Trypanothione disulfide	449.600546	520.67135	483.6402019	215.225433	260.671426	173.45641	77.0090948	12.94970467	74.90654247	94.78138108	-2.155675522	down
(S)-3-Hydroxy-3-methylglutaryl-CoA	177.232668	129.06082	84.63818052	127.004179	185.12245	241.81923	263.501841	214.2476745	284.4143916	245.4361136	0.82954143	up
Oxidized glutathione	12635.6986	13559.261	9044.95337	10845.093	10162.4319	29201.365	29365.6318	42097.40122	25719.5265	28354.17556	1.459969185	up
1-Octadecanoyl-sn-glycero-3-phosphoethanolamine	49801.6288	71547.883	70455.10621	63203.2828	63886.6104	93806.385	119347.328	125730.0326	110424.4933	103979.7999	0.794951469	up
Glutathione	241571.824	217347.51	229360.4259	219576.659	208195.831	73846.841	62037.7983	56367.26825	79918.78398	94659.99866	-1.605218324	down
gamma-L-Glutamyl-L-cysteine	2507.27974	2115.7851	1634.465042	2130.21213	2860.59093	462.12826	1911.91858	1125.152465	1264.635747	1271.844826	-0.898123009	down
20-HETE	19219.7138	19783.723	19918.70576	17056.8023	20727.7523	4393.2196	5719.7032	2637.716143	9551.218758	9052.688625	-1.624941153	down
5(S)-HETE	315.582322	282.76874	448.8581938	424.136556	764.532476	1383.9885	1759.12754	2517.768329	1623.934762	2336.839387	2.105443945	up
20-Hydroxyleukotriene E4	349.33505	352.75313	414.3231435	303.723217	326.971857	2170.5464	946.463433	1086.526081	1281.502999	714.7820142	1.827259084	up
6-Keto-PGF1α	3818.13172	27770.668	3970.985471	5470.86256	6036.42597	59431.277	45204.2826	44990.75254	61347.22904	55615.60517	2.501827983	up
Prostaglandin J2	335.377768	208.35649	316.2993707	342.619043	378.81264	2063.7914	3288.77591	6283.686215	2281.235127	3551.92027	3.465496986	up
11-Dehydro-thromboxane B2	1004.31399	1274.4793	1149.710771	1038.97251	1068.96861	3603.0706	3555.46124	2788.48815	2262.118745	1466.25453	1.304550451	up
2,3-Dinor-8-iso prostaglandin F1alpha	995.468046	826.63047	972.5083278	827.242114	805.24338	1047.3014	1107.36532	1090.935155	1107.054717	1124.620149	0.307099301	up
15-OxoETE	10521.9941	8081.2018	4393.430697	4896.14324	4142.74294	9999.1265	21884.0072	16848.22837	21595.23871	10066.08352	1.327392141	up
12-OxoETE	72224.2581	82474.737	76648.17356	136293.609	116828.465	24915.025	27916.8722	19669.33825	13989.25166	15264.40483	-2.251306922	down
20-Hydroxyeicosatetraenoic	102656.003	80257.931	132039.0988	107218.485	110968.016	212626.09	218701.565	148609.4759	197543.7045	162274.6938	0.817772337	up
Prostaglandin F2alpha	2409.56402	2804.599	7025.126669	2714.3822	2771.9152	779.65985	453.506728	174.6450312	1266.882256	1671.82017	-2.027902709	down
Leukotriene F4	3644.45511	2494.6331	3554.796699	1981.63302	2543.87652	6771.4929	10263.7735	12844.31458	10185.39507	9466.079924	1.800473349	up
17-Hydroxylinolenic acid	1.09477625	1.36E-09	12.17340867	0.48501731	29.6011672	1274.8173	1091.02676	1220.019141	813.6892713	539.9862633	6.832055113	up
alpha-Hydroxylinoleic acid	1.36E-09	16.54509	21.83051727	1.36E-09	21.0462601	590.79126	605.438931	995.5018136	309.7350058	387.0371238	5.603184793	up

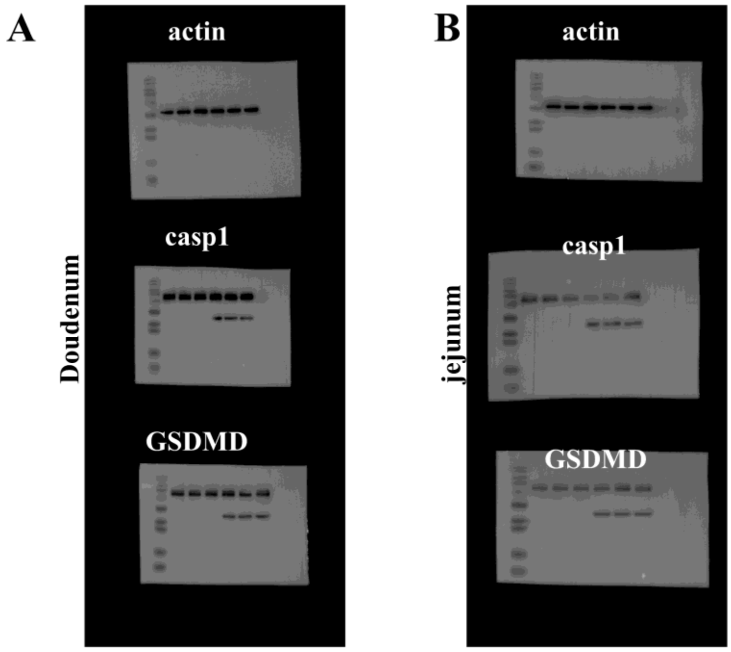

Figure S1: Original Western blot images for Figures 2B and 2C.

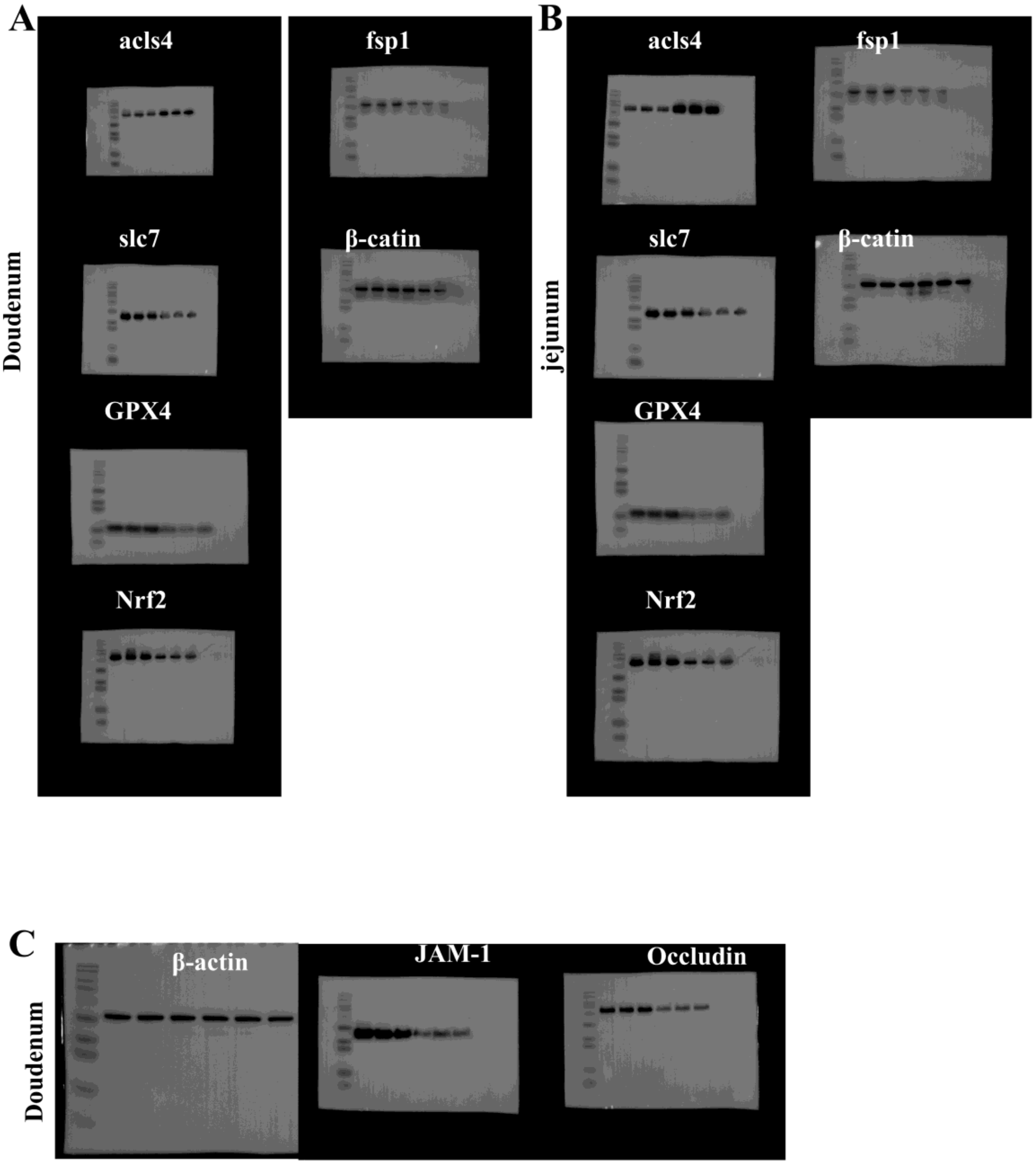

Figure S2: Original Western blot images for Figures 5C, 5E, and 7C.
